# Stepwise genetic testing strategy identified pathogenic variants in 10 Chinese duchenne muscular dystrophy patients

**DOI:** 10.3389/fgene.2026.1805459

**Published:** 2026-06-19

**Authors:** Dengzhi Zhao, Wenke Yang, Ke Yang, Dong Wu, Na Qi, Shixiu Liao

**Affiliations:** Institute of Medical Genetics, Henan Provincial People’s Hospital, People’s Hospital of Zhengzhou University, People’s Hospital of Henan University, Zhengzhou, China

**Keywords:** chromosome inversion, deep intronic variant, Duchenne muscular dystrophy, multiplex ligation-dependent probe amplification, Whole-Exome Sequencing, Whole-Genome Sequencing

## Abstract

**Background:**

Duchenne muscular dystrophy (DMD) results from pathogenic variants in the *DMD* gene. Despite routine screening using Multiplex Ligation-dependent Probe Amplification (MLPA) and Whole-Exome Sequencing (WES), a subset of cases remains molecularly unexplained. This diagnostic gap is often attributed to the inherent limitations of these methods in detecting deep intronic variants or complex structural rearrangements.

**Methods:**

We implemented a tiered molecular diagnostic workflow for 10 male patients with suspected DMD. Primary screening for copy number variations (CNVs) was performed via MLPA. Negative cases were sequentially analyzed using WES and Whole-Genome Sequencing (WGS). Pathogenicity was validated through *in silico* splicing predictions, mRNA transcript analysis, and chromosomal breakpoint mapping.

**Results:**

MLPA identified CNVs in six cases. WES resolved two additional patients, identifying a frameshift variant (c.7392delC) and a nonsense variant (c.4729C>T). In the remaining two cases, WGS identified a deep intronic variant (c.9225–287C>A) and a 9.4 Mb chromosomal inversion. Functional analysis of the c.9225–287C>A variant revealed the activation of a 58-nucleotide pseudoexon, resulting in a frameshift (p.H3076Vfs*15) and truncated dystrophin expression. For the 9.4 Mb inversion, WGS successfully mapped the breakpoints to DMD intron 7, enabling definitive carrier identification within the family.

**Conclusion:**

Our findings demonstrate that WGS is a robust tool for detecting pathogenic variations that evade standard MLPA and WES protocols, including deep intronic variants and large-scale inversions. This study emphasizes the clinical necessity of an integrated, tiered genomic approach to ensure accurate genetic counseling and facilitate access to precision therapies.

## 1 Background

Duchenne muscular dystrophy (DMD) is a severe, X-linked recessive neuromuscular disorder caused by variants in the *DMD* gene, which encodes the essential structural protein dystrophin (Echigoya et al., 2019; Guilbaud et al., 2018; Yu et al., 2017). Affecting approximately one in 3,500 live male births, it is the most common muscular dystrophy diagnosed in childhood (Crispi and Matsakas, 2018). Pathogenic variants in the *DMD* gene, located at the Xp21.2-p21.3 locus, result in a deficiency of dystrophin, leading to progressive muscle degeneration and significantly elevated serum creatine kinase levels (Awano et al., 2024). Clinically, patients typically present with limb weakness between ages 3 and 5, lose ambulation by age 12, and often succumb to respiratory or cardiac failure in their third or fourth decade (Van Ruiten et al., 2016). Beyond physical symptoms, some individuals also experience cognitive impairment (Vaillend et al., 2025). This debilitating clinical progression imposes a substantial burden on both affected families and global healthcare systems.

In recent years, the clinical management of DMD has transitioned from purely symptomatic support to precision intervention. Accurate identification of specific *DMD* gene variants serves as the cornerstone of this paradigm shift (Fang et al., 2025; Fratter et al., 2020). First, molecular diagnosis is essential for precise genetic counseling, enabling carriers to utilize preimplantation genetic testing or prenatal diagnosis to prevent familial disease transmission (Ma et al., 2018; Xu et al., 2020). Second, the rapid advancement of targeted therapies, such as exon-skipping antisense oligonucleotides, nonsense variant read-through agents, and gene-editing technologies, demonstrates strict dependency on variant types (Echigoya et al., 2019; Crispi and Matsakas, 2018; Sun et al., 2020; Sheikh and Yokota, 2021). For example, the FDA-approved eteplirsen (Exondys 51) is indicated exclusively for patients amenable to exon 51 skipping, whereas ataluren targets nonsense variants (Elhawary et al., 2018). Conversely, patients with complex structural rearrangements or deep intronic variants may require emerging strategies such as CRISPR-Cas9-mediated gene correction (Wang et al., 2017; Erkut and Yokota, 2022). Ultimately, definitive variant characterization is imperative to ensure patients are not excluded from life-altering therapeutic opportunities.

**FIGURE 1:**
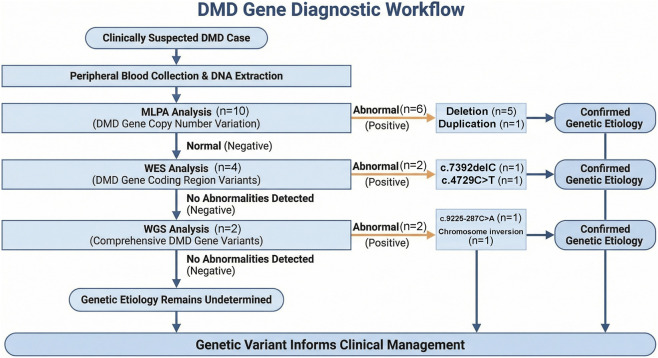
A stepwise flowchart for detecting the genetic causes of DMD.

The *DMD* gene, spanning 2.22 Mb and comprising 79 exons, is one of the largest in the human genome, resulting in a highly diverse mutational spectrum (Zinina et al., 2022; Okubo, 2025; Keegan, 2020). Large-scale exon deletions and duplications account for 70%–80% of cases, while the remainder involves small indels, single-nucleotide variants, and complex structural rearrangements (Wang et al., 2019; Cohen et al., 2022). Identifying these variations is critical for accurate prognosis, carrier screening, and the implementation of targeted therapies (Fortunato et al., 2021). However, individual detection techniques often lack the breadth to capture this full spectrum. Multiplex Ligation-dependent Probe Amplification (MLPA), the current clinical standard, is restricted to detecting copy number variations (CNVs) (Wang et al., 2019). Conversely, Whole-Exome Sequencing (WES) targets coding regions but frequently misses deep intronic variants and large structural variations, while Whole-Genome Sequencing (WGS), though comprehensive, is often hindered by high costs and prolonged turnaround times (Fratter et al., 2020; Wang et al., 2019). To address the need for high detection rates and cost-effectiveness in clinical settings, we employed a stepwise genetic testing strategy to identify pathogenic variants in 10 DMD cases, providing genetic evidence for clinical management of the DMD family. The flowchart illustrating the screening process for genetic variations is shown in Figure 1.

## 2 Materials and methods

### 2.1 Study population

Ten male probands from unrelated families were recruited from the Department of Genetic Diseases at Henan Provincial People’s Hospital between June 2023 and August 2025. The inclusion criteria were: (1) onset of progressive limb weakness between 3 and 5 years of age, (2) serum creatine kinase levels >5,000 U/L, (3) electromyography or muscle biopsy findings consistent with dystrophic changes, and (4) written informed consent for genetic testing and gene therapy assessment. Exclusion criteria included: (1) diagnosis of non-dystrophic myopathies, or (2) incomplete clinical data or loss to follow-up. This study was approved by the Ethics Committee of Henan Provincial People’s Hospital. All participants’ data were anonymized, and written informed consent was obtained from the legal guardians of all patients.

### 2.2 Genetic analysis

#### 2.2.1 Multiplex ligation-dependent probe amplification

Genomic DNA was extracted from peripheral blood using a DNA Extraction Kit (Tiangen, Beijing, China). MLPA was performed utilizing the SALSA MLPA P034/P035 DMD kits (MRC-Holland, Amsterdam, Netherlands), which target the promoter region and all 79 exons of the DMD gene. Probe ligation, amplification, and capillary electrophoresis were conducted on an ABI-3500 Genetic Analyzer (Applied Biosystems, USA) according to the manufacturer’s protocols. Data were analyzed using Coffalyser.Net software (MRC-Holland), with exon copy numbers <1 or >1 (normalized to reference) defined as deletions or duplications, respectively.

#### 2.2.2 Whole-exome sequencing

For MLPA-negative cases, WES was performed on a NovaSeq 6000 platform (Illumina, CA, USA) using the SureSelect Human All Exon V7 Kit (Agilent Technologies, CA, USA). Captured exonic regions were sequenced to an average depth of >100 times, yielding a minimum of 10 Gb of raw data. Quality control was performed using FastQC (v0.11.9). Reads were aligned to the hg19 human reference genome using BWA-MEM (v0.7.17), and variant calling was executed via GATK (v4.2.5.0). Variants were filtered to exclude those with a minor allele frequency >0.01 in the East Asian population within the gnomAD database. Pathogenicity was classified according to the 2015 ACMG/AMP guidelines (Richards et al., 2015). Missense variant impact was predicted using SIFT (http://sift-dna.org/) and PolyPhen-2 (http://genetics.bwh.harvard.edu/pph2/). Candidate pathogenic variants were validated using Sanger sequencing.

#### 2.2.3 Whole-genome sequencing

WGS was applied to patients who tested negative via both MLPA and WES. Libraries were constructed using the TruSeq DNA PCR-Free Library Prep Kit (Illumina) with 350 bp inserts and sequenced on the NovaSeq 6000 platform. Data were aligned to the GRCh38/hg38 reference genome. Deep intronic variants (defined as >200 bp from exon–intron boundaries) were analyzed for splicing effects using SpliceAI (https://spliceailookup.broadinstitute.org/) and Human Splicing Finder (http://www.umd.be/HSF/). To verify splice-site pathogenicity, RNA was extracted from muscle tissue for transcript analysis. Structural variants were detected using SpeedSeq with default parameters (Chiang et al., 2015). Chromosomal inversion breakpoints were validated via PCR and Sanger sequencing.

### 2.3 Sanger sequencing

All single-nucleotide variants were confirmed via Sanger sequencing. Genomic DNA was isolated from peripheral blood using a DNA Extraction Kit (Tiangen, Beijing, China). Specific primers flanking each candidate variant were designed and synthesized by Tsingke Biological Technology (Zhengzhou, China). PCR amplification was performed using an S1000 Thermal Cycler (Bio-Rad, CA, USA), and the resulting products were sequenced by Tsingke Biological Technology (Zhengzhou, China).

## 3 Results

### 3.1 Baseline clinical characteristics

The study cohort comprised 10 male patients with a mean age of onset of 3.5 years. The mean serum creatine kinase level was 10,800 U/L. While all patients initially presented with lower limb weakness, two also exhibited concurrent upper limb weakness, and one presented with a mild intellectual disability. Regarding inheritance, three patients had a positive family history, while the remaining seven were sporadic cases. Clinical features of all included patients are summarized in Table 1. Family pedigrees are shown in Supplementary Figure S1.

**TABLE 1:** Clinical features of included patients with suspected Duchenne muscular dystrophy.

Patient ID	Gender	Age at onset (year)	Creatine kinase (U/L)	Phenotype	Family history
P-1	Male	1.7	12,737	Lower limb weakness, difficulty in walking, hypotonia, growth retardation	Positive
P-2	Male	5.6	6,017	Lower and upper limb weakness, delayed movement, gowers sign, gastrocnemius hypertrophy	Negative
P-3	Male	1.8	10,605	Lower limb weakness, difficulty in walking, hypotonia, gross motor developmental delay	Positive
P-4	Male	3.2	26,902	Lower limb weakness, gastrocnemius hypertrophy	Negative
P-5	Male	5.3	11,500	Lower and upper limb weakness, delayed movement, difficulty in walking, gowers sign, gastrocnemius hypertrophy	Negative
P-6	Male	2.7	10,214	Lower limb weakness, difficulty in walking, gowers sign, gastrocnemius hypertrophy	Positive
P-7	Male	4.9	7,320	Lower limb weakness, gastrocnemius hypertrophy	Negative
P-8	Male	2.1	11,143	Lower limb weakness, gowers sign, gross motor developmental delay, gastrocnemius hypertrophy	Negative
P-9	Male	5.4	4,121	Lower limb weakness, gastrocnemius hypertrophy, mild intellectual disability	Negative
P-10	Male	2.3	7,442	Lower limb weakness, gross motor developmental delay	Negative

### 3.2 MLPA analysis for *DMD* deletions and duplications

**FIGURE 2:**
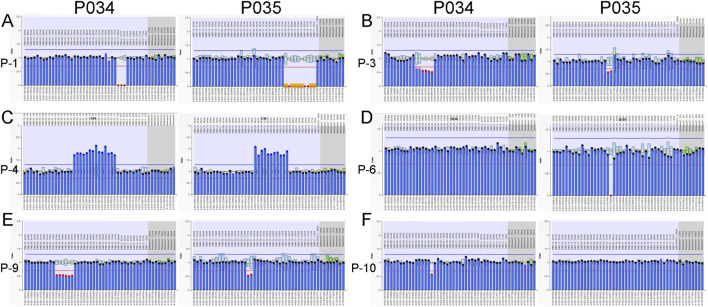
MLPA results for *DMD* exon copy numbers of patients. Data were obtained using the SALSA MLPA P034/P035 DMD kit for **(A)** P-1, **(B)** P-3, **(C)** P-4, **(D)** P-6, **(E)** P-9, and **(F)** P-10.

All 10 patients underwent MLPA to identify CNVs in the *DMD* gene. The CNV distributions are summarized in Table 2. MLPA identified CNVs in six patients P-1, P-3, P-6, P-9, and P-10 exhibited exon deletions, while patient P-4 showed an exon duplication. Representative MLPA results are illustrated in Figure 2. No copy number differences were detected in patients P-2, P-5, P-7, and P-8; consequently, next-generation resequencing was employed to screen for single-nucleotide variants in these individuals.

**TABLE 2:** MLPA analysis of *DMD* exon copy numbers in the patients.

Patient ID	Copy number	Exons affected
P-1	Deletion	8–20
P-2	Normal	NA
P-3	Deletion	45–52
P-4	Duplication	20–44
P-5	Normal	NA
P-6	Deletion	51
P-7	Normal	NA
P-8	Normal	NA
P-9	Deletion	45–52
P-10	Deletion	45

NA, not applicable.

### 3.3 Identification of pathogenic *DMD* variants c.7392delC and c.4729C>T via WES

**FIGURE 3:**
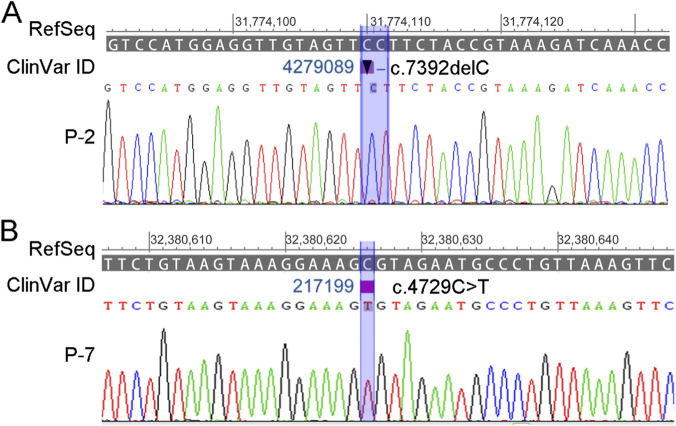
Sanger sequencing validation of identified *DMD* variants. **(A)** Sanger sequencing electropherogram of the c.7392delC variant in patient P-2. **(B)** Sanger sequencing electropherogram of the c.4729C>T variant in patient P-7.

WES was conducted for the four patients (P-2, P-5, P-7, and P-8) who yielded negative MLPA results. This analysis identified pathogenic exonic variants in two cases. Specifically, patient P-2 harbored a hemizygous frameshift variant, c.7392delC (p.Ser2464_Leu2465insTer), in the *DMD* transcript (NM_004006.3). Patient P-7 carried a hemizygous nonsense variant, c.4729C>T (p.Arg1577Ter). Both variants were subsequently validated using Sanger sequencing (Figure 3). These variants are documented in the ClinVar database (Variation IDs: 4279089 and 217199, respectively) and are classified as likely pathogenic and pathogenic, respectively. In contrast, no known or potentially pathogenic *DMD* variants were identified in patients P-5 or P-8.

### 3.4 Identification and functional characterization of a pathogenic deep intronic *DMD* variant inducing pseudoexon activation

**FIGURE 4:**
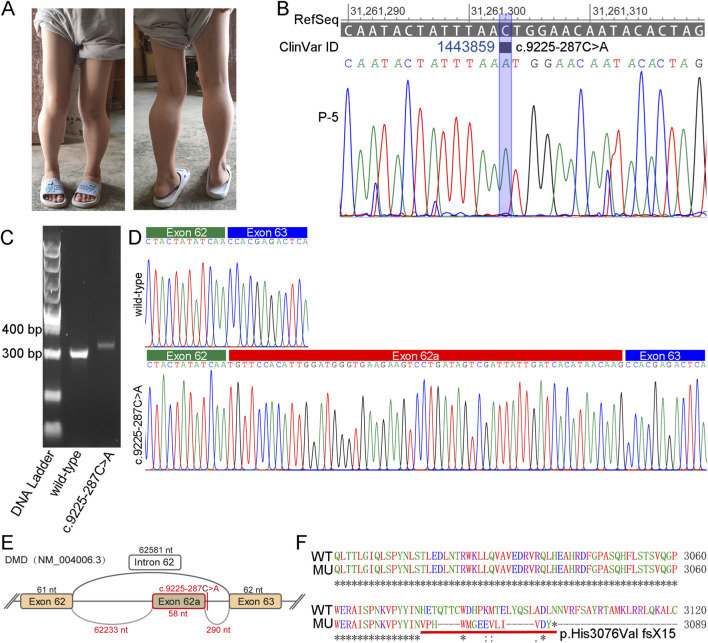
Functional characterization of the deep intronic *DMD* variant c.9225-287C>A. **(A)** Anterior and posterior views of the lower limbs of patient P-5, illustrating gastrocnemius hypertrophy. **(B)** Sanger sequencing confirmation of the c.9225-287C>A variant in patient P-5. **(C)** Reverse transcription PCR analysis showing the impact of the c.9225-287C>A variant on *DMD* transcript splicing product. **(D)** Sanger sequencing confirms the sequence of the pseudoexon 62a. **(E)** Schematic representation of the splicing alterations induced by the c.9225-287C>A variant. **(F)** Comparison of amino acid sequences translated from normal and aberrant splicing products. The c.9225-287C>A variant activates a pseudoexon, resulting in the truncated protein p.H3076Vfs*15.

WGS of patient P-5 identified a deep intronic variant, c.9225–287C>A, in the *DMD* gene (NM_004006.3), which was subsequently validated via Sanger sequencing (Figures 4A,B). Although previously reported (Okubo et al., 2017), its pathogenicity had not been evaluated, and it remains classified as a variant of uncertain significance in the ClinVar database (ID: 1443859). SpliceAI and HSF algorithms predicted that this variant creates a cryptic splice donor site (SpliceAI donor gain score: 0.84; HSF Matrix score: 102.14) located 3 bp downstream of the variant.

Histopathological examination of the muscle biopsies from patient P-5 revealed myopathic atrophy of muscle fibers (Supplementary Figure S2). To characterize the functional impact of c.9225-287C>A, *DMD* transcript analysis was performed on muscle tissue. Following RNA extraction and reverse transcription, agarose gel electrophoresis demonstrated that the mutant amplicon was larger than the wild-type (Figure 4C). Sanger sequencing further confirmed that the mutant transcript incorporated a 58-nucleotide pseudoexon (designated 62a), as illustrated in the schematic (Figures 4D,E). This insertion disrupts the open reading frame (p.H3076Vfs*15) and introduces a premature termination codon, resulting in a truncated, non-functional protein (Figure 4F). These findings establish the pathogenic role of the c.9225-287C>A variant in Duchenne muscular dystrophy.

### 3.5 Identification and breakpoint mapping of a 9.4 Mb inversion disrupting the *DMD* gene

**FIGURE 5:**
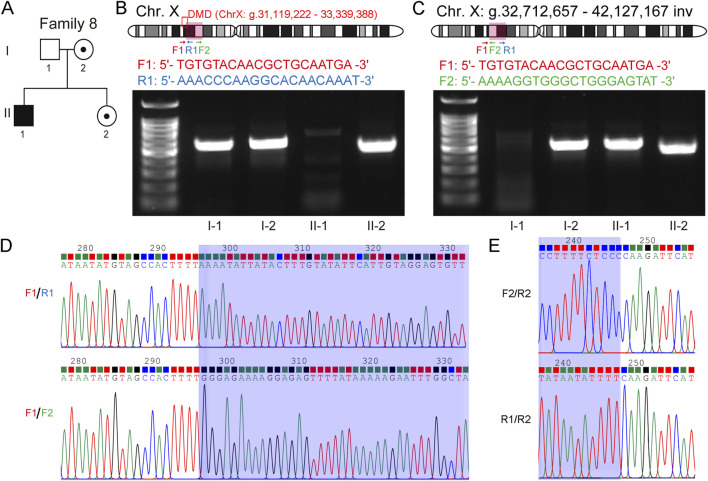
Characterization of the *DMD* breakpoint in Family 8. **(A)** Pedigree of Family 8, including the clinically diagnosed DMD patient P-8 (II-1). **(B,C)** Schematic of the chromosomal inversion region detected by WGS. The red box above the chromosome schematic represents the genomic localization of the *DMD* gene in the reference genome (ChrX: g.31119222-33339388, GRCh38). The pink-purple shaded area covering the chromosome schematic represents the inversion variant region identified by WGS (ChrX: g.32712657-42127167 inv, GRCh38). The red, blue, and green arrows below the chromosome schematic indicate the relative positions of the PCR primers F1, R1, and F2 used for amplifying the flanking regions of the *DMD* breakpoint (upper panel). Primer sequences used to determine the breakpoint by PCR (middle panel). Agarose gel electrophoresis of PCR products (lower panel). **(D)** Sanger sequencing chromatograms confirming the *DMD* breakpoint junction. **(E)** Sanger sequencing confirmed the *CASK-PPP1R2C* intergenic region breakpoint junction at coordinates chrX: 42127167. The sequences of the PCR primers F2, R2, and R1 are 5′-AAA​AGG​TGG​GCT​GGG​AGT​AT-3′, 5′-GGA​CAC​CTG​GAC​TGG​TCA​TT-3′, and 5′-AAA​CCC​AAG​GCA​CAA​CAA​AT-3′, respectively.

WGS of patient P-8 identified a hemizygous inversion spanning approximately 9.4 Mb on chromosome X (ChrX: g.32712657-42127167 inv, GRCh38). The two breakpoints were localized to intron 7 of the *DMD* gene and the *CASK-PPP1R2C* intergenic region. Notably, this large-scale chromosome inversion was not detected by conventional karyotyping with a resolution of 550 bands. Pedigree analysis after agarose gel electrophoresis of PCR amplification products demonstrated that the variant was inherited from the mother (I-2) and that the patient’s younger sister is a carrier (Figures 5A–C). Furthermore, Sanger sequencing confirmed the *DMD* breakpoint junction at coordinates chrX:32712657 (Figure 5D). The breakpoint in the *CASK-PPP1R2C* intergenic region was also confirmed (Figure 5E). For this family, the precise mapping of these inversion breakpoints provides a definitive molecular diagnosis and a critical framework for genetic counseling, reproductive selection, and personalized clinical management.

## 4 Discussion

This study underscores the diagnostic necessity of a tiered genomic approach, integrating MLPA, WES, and WGS, to resolve the molecular etiology of DMD. We characterized the genetic basis of 10 unrelated Chinese patients: MLPA identified pathogenic variants in six individuals, WES identified coding-region variants in two patients, and WGS resolved the remaining two cases involving a deep intronic variant and a large chromosomal inversion.

MLPA remains the recommended first-line test due to its ability to efficiently detect common exon CNVs at minimal cost (Fratter et al., 2020; Wang et al., 2019). In this cohort, MLPA achieved a 60% diagnostic yield, highlighting its utility for primary care facilities with limited resources. Beyond diagnosis, these results directly inform therapeutic eligibility. For example, a patient with an exon 51 deletion is a candidate for eteplirsen (Exondys 51), while a patient with a 45–50 deletion may qualify for other clinical trials (Elhawary et al., 2018; Wang et al., 2017; Erkut and Yokota, 2022). However, MLPA cannot detect single-nucleotide variants or intronic variants, necessitating further analysis for negative cases.

WES addresses this gap by identifying coding-region single-nucleotide variants and small indels (Yang et al., 2013). Beyond confirming pathogenicity for nonsense and missense variants, WES facilitates precise genetic counseling and reproductive options, such as preimplantation genetic testing. Therapeutically, identifying nonsense variants allows for the evaluation of read-through agents like ataluren (Elhawary et al., 2018). Additionally, WES enables panel-free screening, ruling out other myopathies that clinically mimic DMD. While WES balances cost and utility, it remains blind to deep intronic and structural variants that are increasingly relevant for emerging gene-editing technologies.

WGS serves as the definitive tier for MLPA/WES-negative cases. In this study, WGS identified two complex variants, a deep intronic splice-site variant and a 9.4 Mb X-chromosome inversion. The identification of the intronic variant c.9225–287C>A is particularly significant; although previously classified as a variant of uncertain significance, our functional analysis confirmed that it activates a 58-nucleotide pseudoexon 62a. The insertion of this pseudoexon disrupts the sequential grouping of nucleotide triplets into codons, resulting in a frameshift and a premature termination codon (PTC). This frameshift and the subsequent PTC likely trigger nonsense-mediated mRNA decay, leading to the degradation of the aberrant transcript. If translated, the resulting truncated protein (p.H3076Vfs*15) would lack critical functional domains, producing non-functional dystrophin and a subsequent loss of sarcolemmal stability, which is the hallmark of DMD pathology. Furthermore, WGS identified a 9.4 Mb inversion disrupting intron 7 of *DMD* at the single-nucleotide level (breakpoint at chrX: 32712657), which was further validated by Sanger sequencing. In this context, WGS demonstrated significant superiority over both conventional and high-resolution karyotype analysis. Precise mapping of breakpoints via WGS is indispensable for carrier testing and the design of CRISPR-Cas9-mediated correction strategies (Wang et al., 2017; Erkut and Yokota, 2022). Despite higher costs, WGS provides irreplaceable therapeutic value for complex cases and prevents misdiagnosis by screening for concurrent variants in other myopathy-related genes.

Our diagnostic strategy employs a tiered, stepwise approach to maximize yield while minimizing costs and analytical burden. MLPA is a rapid, cost-effective, and highly accurate technique for detecting exon-level deletions and duplications, which account for approximately 70%–80% of DMD cases (Wang et al., 2019; Cohen et al., 2022). By prioritizing MLPA, the majority of patients can be efficiently diagnosed without the need for more expensive and complex next-generation sequencing (NGS). For MLPA-negative cases, we proceed to WES or WGS, which effectively identify pathogenic variants, including single-nucleotide variants, small indels, and chromosomal inversions, covering the remaining 20%–30% of cases. Although WGS could theoretically replace both MLPA and WES, advocating for its use as a universal first-tier test remains challenging due to significant global disparities in healthcare infrastructure, funding, and technical expertise. The implementation of a robust NGS diagnostic program requires substantial capital investment in sequencing platforms, high-performance computing for data analysis, and a specialized bioinformatics workforce resources often unavailable in many regions (Yin et al., 2021; Rehder et al., 2021). Furthermore, the interpretation of WGS data, particularly non-coding and deep intronic variants of uncertain significance, poses a considerable challenge requiring sophisticated clinical correlation and functional studies (Rehder et al., 2021). Therefore, while WGS represents the future of genetic diagnostics, a pragmatic and resource-aware approach is essential for global application. Given the current landscape of DMD genetic diagnosis, MLPA remains a more accessible and economically viable option for the majority of cases. Our workflow applies NGS selectively, ensuring that advanced technologies are utilized where they provide the greatest diagnostic value.

By implementing the three-tiered diagnostic strategy, we achieved a high detection rate while balancing cost-effectiveness and turnaround time. Although the sample size in this study is limited, the workflow systematically identifies pathogenic variants and minimizes technical blind spots across the full spectrum detectable by these methodologies. Future studies with larger cohorts are warranted to further validate the clinical applicability of this approach. Moreover, as sequencing costs continue to decline, the clinical utility of WGS will likely expand, eventually facilitating a one-stop diagnostic method to improve efficiency and personalize clinical management for DMD families worldwide.

## Data Availability

The datasets presented in this article are not readily available because of ethical/privacy restrictions. Requests to access the datasets should be directed to the corresponding authors.

## Supplementary material

SUPPLEMENTARY FIGURE S1 Pedigrees of ten families with DMD phenotype.

SUPPLEMENTARY FIGURE S2 Pathological results of patient P-5. **(A)** H&E staining (40×) revealed variation in muscle fiber size, with focal myofiber degeneration, necrosis, and regeneration. **(B)** H&E staining (100×) showed partial muscle fiber atrophy, with altered fiber transparency; fibrous connective tissue proliferation was observed between muscle bundles and individual fibers. **(C)** ATPase staining demonstrated a predominance of type I fibers over type II fibers, with atrophy evident in both fiber types, primarily affecting type I fibers. The abnormal fiber type distribution is indicative of a myopathic process. **(D)** SDH staining revealed patchy hypostaining in partial muscle fibers, suggesting metabolic abnormalities in the fibers.
