# Supplementary figures and images for: Stepwise genetic testing strategy identified pathogenic variants in 10 Chinese duchenne muscular dystrophy patients
Source: Front Genet. 2026 Jun 19;17:1805459. doi: 10.3389/fgene.2026.1805459 (PMC13327659; doi:10.3389/fgene.2026.1805459)

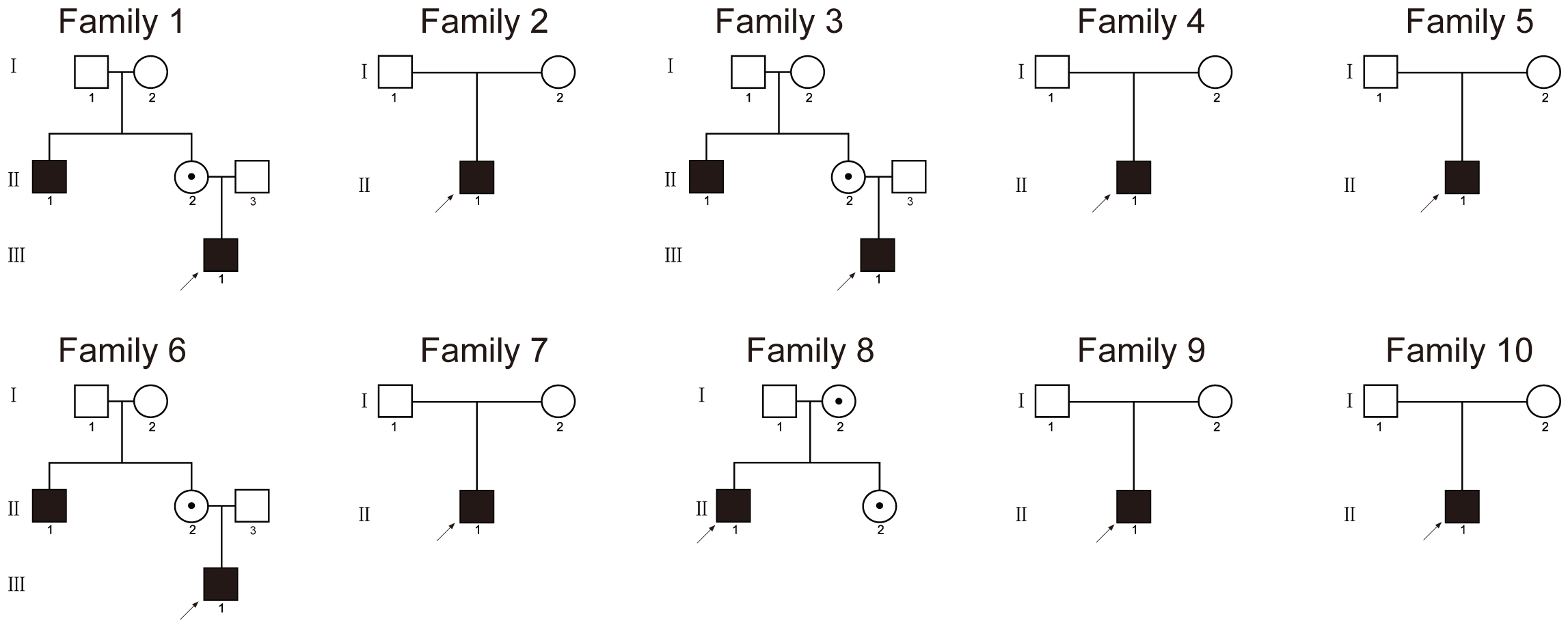

Supplement: Supplementary file 1 [file Image1.jpeg]

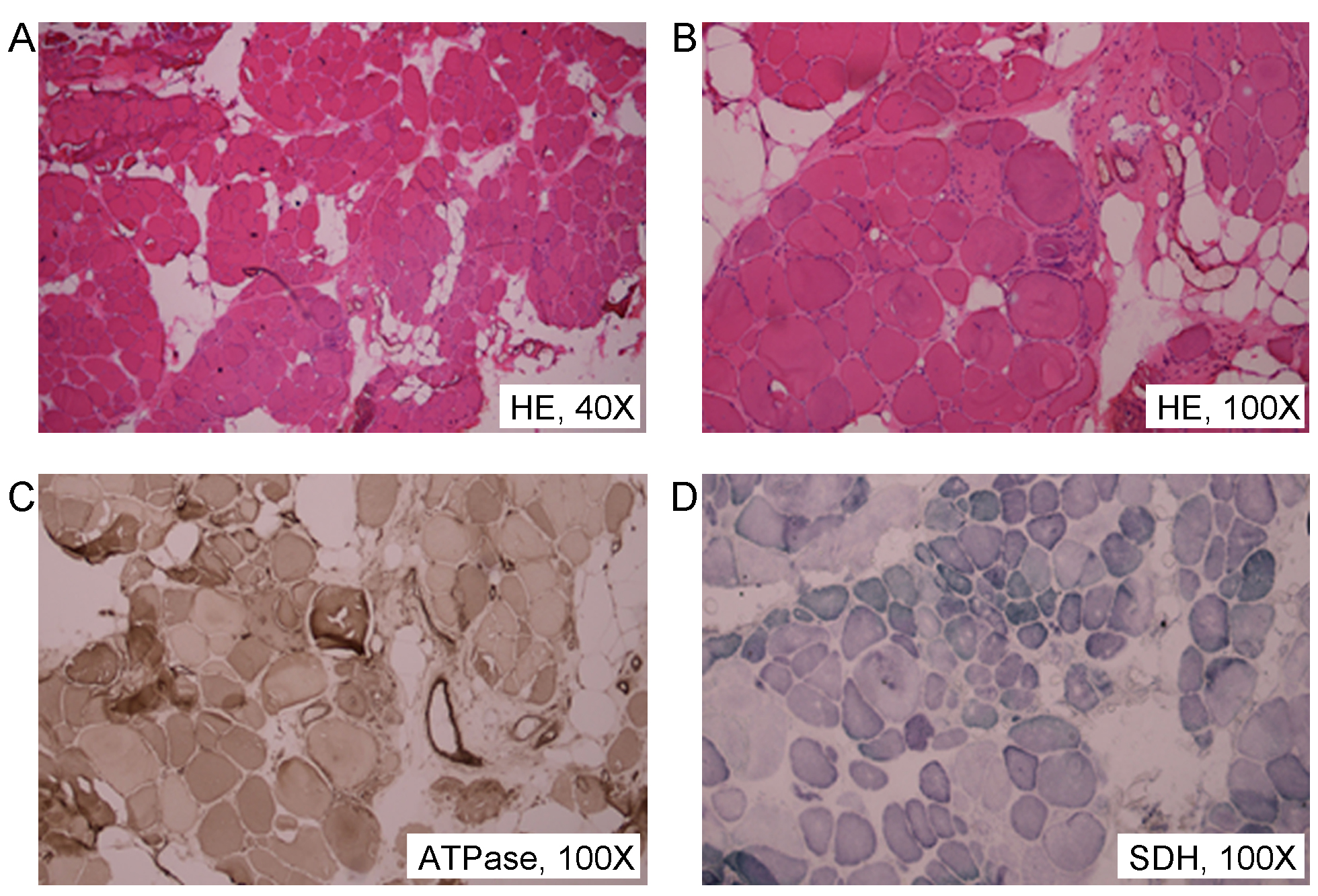

Supplement: Supplementary file 2 [file Image2.jpeg]
